# Molecular Characterization of Methicillin- Resistant *Staphylococcus aureus* in a Tertiary Care hospital in Kuwait

**DOI:** 10.1038/s41598-019-54794-8

**Published:** 2019-12-06

**Authors:** Wadha Alfouzan, Edet E. Udo, Azizah Modhaffer, Asma’a Alosaimi

**Affiliations:** 1 Microbiology Unit, Department of Laboratory Medicine, Farwaniya hospital, Ministry of Health, Sabah Al Nasser, Kuwait; 2 Department of Microbiology, Faculty of Medicine, Kuwait University, Jabriya, Kuwait

**Keywords:** Clinical microbiology, Bacterial infection

## Abstract

Methicillin-resistant *Staphylococcus aureus* (MRSA) are a major cause of healthcare and community- associated infections due to their ability to express a variety of virulence factors. We investigated 209 MRSA isolates obtained from 1 January to 31 December 2016 using a combination of phenotypic and genotypic methods to understand the genetic backgrounds of MRSA strains obtained in a General hospital in Kuwait. Antibiotics susceptibility was performed with disk diffusion, and MIC was measured with Etest strips. Molecular typing was performed using SCC*mec* typing, *spa* typing, and DNA microarray for antibiotic resistance and virulence genes. The isolates were susceptible to vancomycin, teicoplanin, rifampicin, ceftaroline, and linezolid but were resistant to gentamicin, tetracycline, erythromycin, fusidic acid, chloramphenicol and ciprofloxacin. Molecular typing revealed six SCC*mec* types, 56 *spa* types and 16 clonal complexes (CC). The common SCC*mec* types were type IV (39.5%), type III (34.4%), type V (25.8%) and type VI (3.8%). The dominant *spa* types were t860 (23.9%), t945 (8.6%), t127 (6.7%), t688 (6.7%), t304 (6.2) and t044 (5.7%). The other *spa* types occurred sporadically. Genes for PVL was detected in 59 (28.2%) of the isolates. CC8-ST239-MRSA-III + SCCmer (23.3%) was the most prevalent clone, followed by CC6-MRSA-IV (8.3%), CC80-MRSA-IV [PVL+] (5.8%), CC5-MRSA-VI + SCCfus (5.0%), CC30-MRSA-IV[PVL+] (4.1%), CC1-MRSA-V + SCCfus [PVL+] (4.1%), CC5-MRSA-V + SCCfus (4.1%) and CC22-MRSA-IV[PVL+] (4.1%). The study revealed that despite the emergence of MRSA with diverse genetic backgrounds over the years, ST239-MRSA-III remained the dominant clone in the hospital. This warrants reassessment of infection prevention and control procedures at this hospital.

## Introduction

Methicillin-resistant *Staphylococcus aureus* (MRSA) is an opportunistic pathogen that can cause mild to invasive, life-threatening infections^1^, MRSA was first reported in the United Kingdom, soon after the use of methicillin in the healthcare system in the 1960’s^2^, but have since been reported in many countries^3–5^. Methicillin-resistance is mediated by *mecA* gene which encodes penicillin-binding protein 2a (PBP 2a) that results in resistance to beta-lactam antibiotics such as methicillin, cloxacillin and oxacillin^5,6^. The *mecA* gene is located on a mobile genomic island known as staphylococcal cassette chromosome *mec* (SCC*mec*) and is inserted in the chromosome of methicillin-resistant *staphylococci*^5^. The SCC*mec* genetic element differ in size and structural organisation, and on the basis of their size and structural differences, 13 SCC*mec* types designated types I-XIII have been reported^6,7^.

MRSA isolates have been characterised as either hospital or healthcare- hospital-associated MRSA (HA-MRSA) or community-associated (CA-MRSA) on the basis of their SCC*mec* types. HA-MRSA harbour SCC*mec* types I, II and III while the CA-MRSA isolates carry SCC*mec* types IV, V and VI^6,8^. SCC*mec* typing^9,10^, and other molecular typing methods including pulsed-field gel electrophoresis, multilocus sequence typing, Staphylococcal protein A (*Spa*) typing, DNA microarray and whole genome sequencing^5,6,11–13^, have been employed to investigate the clonal distributions of MRSA strains in different countries, and have revealed a diversity in their genetic backgrounds in the different geographical locations.

The epidemiology of MRSA strains is constantly changing and their prevalence and characteristics are known to vary between hospitals in the same country or wards in the same hospital^3,14,15^. Consequently, it is important to study MRSA isolates from local healthcare facilities with unique patient populations to obtain data that can aid empirical treatment of infections and to compare them with those obtained in other healthcare facilities in the country. The Farwaniya Hospital, Kuwait is a university-affiliated hospital with 1,200 beds, which provide services that include medical, surgical, orthopaedic, obstetric and gynaecologic, paediatrics, and two intensive care units^16,17^. A previous study conducted on MRSA obtained at the Farwaniya hospital from 1996 to 2001 using pulsed-field gel electrophoresis, and coagulase gene typing revealed the presence of a dominant and persistent multiresistant MRSA clone^16^, that was later identified as ST239-MRSA-III clone^18^. In addition, the majority of MRSA isolates acquired by patients admitted to the Intensive Care unit of the hospital in 2005–2007 were ST239-MRSA-III^19^. confirming the persistence of ST239-MRSA-III in the hospital. Recent studies on MRSA obtained in other hospitals in Kuwait have revealed the presence of diverse CA-MRSA clones^20,21^. This study was conducted on MRSA isolates obtained from patients admitted to the Farwaniya hospital in 2016 to determine their antibiotic resistance patterns and clonal distribution and compare the results to those obtained in 1996–2001 for the purpose of assisting in patients’ management and control of infections, and to compare the MRSA clones to those in other Kuwait hospitals.

## Results

The 209 isolates were obtained from 209 patients comprising 143 males (68.4%) and 66 females (31.6%) located at the male medical wards (N = 60), male surgical wards (N = 31), female medical wards (N = 4), female surgical wards (N = 4), Obstetrics & gynaecology ward (N = 24), Intensive care units (N = 21), CCU (N = 6), Paediatrics ward (N = 9), Paediatric intensive care unit (6), Casualty (N = 6), Orthopaedics (N = 5), Urology (N = 6), ENT (N = 6) and Out Patient department (N = 13). The location for three patients was not specified.

All of the 209 MRSA isolates were susceptible to rifampicin, linezolid and vancomycin (MIC ≤ 2 µg/ml). The distribution of MIC values for vancomycin and teicoplanin are summarized on Table 1 and the MIC values for each isolates is presented in a Supplementary Table 1. In total, 202 (96.8%) were sensitive to teicoplanin with MIC ≤ 2 µg/ml, six (3.4%) isolates had MIC: 3 µg/ml and one isolate had MIC:4. µg/ml. Most of the isolates were susceptible to vancomycin and teicoplanin with MIC of 1.5 µg/ml.

**Table 1:** MIC values for vancomycin and teicoplanin.

S/N	MIC µg/ml	Vancomycin N (%)	Teicoplanin N (%)
1	≤0.5	6 (2.9)	4 (1.9)
2	0.75	15 (7.2)	10 (4.7)
3	1.0	67 (32.0)	43 (20.5)
4	1.5	69 (33.0)	78 (37.3)
5	2.0	52 (24.8)	67 (32.0)
6	3.0	Nil	6 (2.9)
7	4.0	Nil	1 (0.5)

The isolates were resistant to fusidic acid (N = 133; 63.4%), gentamicin (N = 114; 54.5%), tetracycline (N = 114; 54.5%), erythromycin and clindamycin (N = 109; 52.2%), ciprofloxacin (N = 104; 49.8%) and trimethoprim (N = 45; 21.5%), Thirty (14.4%) and 79 MRSA isolates expressed inducible and constitutive resistance to clindamycin, respectively. Nineteen (9.0%) and four (1.9%) isolates were resistant to chloramphenicol and high-level mupirocin respectively. In total, 164 (78.5%) of the isolates expressed multiresistance (resistance to three or more classes of antibiotics) while 45 (21.5%) isolates were non-multiresistant (resistant to less than three classes of antibiotics (Table 2). Most of the isolates were resistant to six (28.7%), classes of antibiotics. This was followed by isolates that were resistant to five, four and three classes of antibiotics. Nineteen (9.0%) were resistant only to beta-lactam antibiotics (Table 2).

**Table 2:** Antibiotic resistance patterns of Multiresistant isolates.

S/N	# of antibiotic classes	Antibiotic resistance pattern	# of isolates N = 209 (%)
1	8	PG, Gn Km, Em, Clin, Tet, Tp, Cip, Fa, MupH	1 (0.4)
2	7	PG, Gn, Km, Em, Clin, Tet, Tp, Cip, Fa	8 (3.8)
3	6	PG, Gn, Km, Em, Clin, Tet, Tp, Cip	60 (28.7)
4	5	PG, Em, Clin, Cm, Tet, Tp/Fa	32 (15.3)
5	4	PG, Km, Tp, Cip	8 (3.8)
6	4	PG, Gn, Km, Tet, Fa	24 (11.5)
7	3	PG, Km, Em, Clin	18 (8.6)
8	3	Gm, Km, Fa	13 (6.2)
9	2	PG, Tp	9 (4.3)
10	2	PG Km	17 (8.1)
12	1	PG	19 (9.0)

**Abbreviations:** PG, Penicillin G, Hn, gentamicin, Km, kanamycin, Em, erythromycin, Clin, clindamycin, Tet, tetracycline, Tp, trimethoprim, Cip, ciprofloxacin, MupH, high-level mupirocin.

### Molecular characterization of MRSA in isolates

SCC*mec* typing revealed the presence of six SCC*mec* types with the majority belonging to SCC*mec* type IV (N = 75; 39.5%) followed by SCC*mec* types III (N = 72; 34.4%), SCC*mec* type V (N = 52; 25.8%), SCC*mec* type V1 (N = 5; 2.4%), SCC*mec* type I (N = 2; 1%) and SCC*mec* type II (N = 1;0.5%).

*Spa* typing of the 209 isolates revealed 56 *spa* types. *Spa* type t860 was the dominant *spa* type and was detected in 50 (23.9%) of the isolates. This was followed by t945 (N = 18; 8.6%), t127 (N = 14; 6.7%), t688 (N = 14; 6.7%), t304 (N = 13; 6.2%) and t044 (12; 5.7%). The *spa* types that occurred sporadically were t003, t002, t005, t008, t018, t019, t021, t032, t037, t042, t045, t148, t105, t1120, t11836, t14700, t1247, t12398, t16185, t1839, t16202, t148, t306, t314, t355, t359, t362, t363, t376, t3841, t425, t535, t5414, t657, t6845, t690, t701, t713, t7583, t790, t8154, t8168, t852, and t084. The *spa* gene could not be amplified in three isolates.

### DNA microarray analysis of MRSA isolates

A total of 120 MRSA isolates representing the 56 *spa* types were selected for DNA microarray analysis. The selection was based on their *spa* types, and clinical samples. Care was taken to include different *spa* types from all clinical samples. The results yielded 16 clonal complexes (CCs) and one singleton. The distribution of MRSA clonal complexes and their genotypes are shown in Supplementary Table 2). The most prevalent CC was CC8/ST239 (N = 32; 26.6%) followed by CC5 (21; 17.5%), CC1 (11; 9.1%), CC6 (11; 9.1%), CC22 (11; 9.1%), CC80 (8; 6.6%) and CC30 (7; 5.8%), CC97 (N = 4; 3.3%), CC15 (N = 3; 2.5%), CC152 (N = 2; 1.6%), CC88 (N = 2; 1.6%), CC361 (N = 2; 1.6%), CC2250/2277 (N = 1;0.8%), CC45 (N = 1; 0.8%), CC121 (N = 1; 0.8%), CC59 (N = 1;0.8%) and a singleton ST2867.

Thirty of the 32 CC8 isolates belonged to ST239-MRSA-III + SCCmer, (N = 28), ST239-MRSA-III + ccrC (N = 1) and ST239-MRSA-III + SCCmer + ccrC (N = 1) clone making ST239-MRSA-III + SCCmer the commonest genotype. The ST239-MRSA-III isolates belonged to six *spa* types consisting of t860 (N = 16), t945 N = 10,) and one each of t037, t713, t425 and t1247. The other common genotypes were CC6-MRSA-IV, WA MRSA-51 (N = 10), CC80-MRSA-IV[PVL^+^] European CA-MRSA clone (N = 7), CC5-MRSA-VI + SCCfus (N = 6), CC5-MRSA-V + SCCfus, WA MRSA-14/109 (N = 5), CC1-MRSAV + SCCfus [PVL^+^] (N = 5), CC22-MRSA-IV[PVL+] (N = 5) and CC30-MRSA-IV[PVL^+^] (N = 5). The remaining genotypes occurred in one to four isolates.

### Distribution of MRSA isolates among clinical sources

We examined MRSA isolates analysed by DNA microarray according to their clinical sources to establish any specific association between clones and clinical sources and infection types. The most common clinical samples that yielded MRSA isolates were wound (N = 52), blood (N = 26), pus (N = 22), and the respiratory samples, sputum (N = 5) and tracheal aspirates (N = 13). The other specimens yielded fewer MRSA isolates. The well-known HA-MRSA clone, ST239-MRSA-III, was the dominant isolate in axilla, groin, and was the major isolate in nasal and wound samples but was isolated sporadically from blood, skin and respiratory samples (sputum and tracheal aspirates). In contrast, blood, wound and respiratory samples yielded higher proportions of diverse clones carrying CA-MRSA genotypes (that is carrying SCC*mec* IV, V, VI) with none of the clones dominating in the clinical samples.

### Antibiotic resistance genotypes

All isolates were resistant to benzyl penicillin and harboured *blaZ* and *mecA*. All of the gentamicin- resistant isolates were positive for *aacA-aphD* that encodes resistance to gentamicin, kanamycin, amikacin and tobramycin, and *aphA3* which mediates resistance to kanamycin and neomycin. The *aphA3* was also detected in two isolates of CC361 (n = 1) and CC59 (N = 1) that were resistant to kanamycin but susceptible to gentamicin. Fifty isolates, belonging to CC8-ST239 (n = 29), CC6 (N = 10), CC22 (7), CC15 (N = 3), and CC5 (N = 1), harboured *aadD* which encodes resistance to kanamycin, neomycin and tobramycin.

The fusidic acid-resistant isolates were positive for *fusC* (N = 26) or *fusB* (N = 10). While *fusB* was only detected in isolates of CC80 (N = 7) and CC5 (N = 3), *fusC* was detected in isolates with diverse genetic backgrounds.

Erythromycin resistance was associated with different resistance determinants including *ermA* (rRNA methyltransferase A), *ermC* (rRNA methyltransferase C), *linA* (lincosaminide nucleotidyltransferase), *msr(A)* (macrolide efflux pump) and *mph(C)* (macrolide phosphotransferase II). *ermA* was the most prevalent erythromycin resistance determinant. It was detected in 28 ST239 and one CC5 isolates. This was followed by *ermC* which was detected in 26 isolates of diverse genetic backgrounds. *ermB* was detected in a single CC59 isolate. Both *mph(C)* and *msr(A)* occurred together in eight isolates of CC1-ST772-MRSA- (N = 1), CC1-MRSA-V + SCCfus (N = 2), CC8-MRSA-IV (N = 1), CC5-MRSA-IV-Paediatric clone (N = 1), CC30-MRSA-IV[PVL+] (N = 2) and CC361-MRSA-IV (N = 1). *linA* was detected in three CC15 and one ST239 isolates. A single CC97 isolate was positive for *vga(A*), an ABC transporter that confers resistance to streptogramins.

Tetracycline resistance was detected in 55 of the 120 isolates analysed by microarray. The tetracycline resistance determinants, *tetK* and *tetM*, were detected in 50 of the 55 isolates respectively. Five CC1-MRSA-V+ SCCfus [PVL+] isolates were negative for both *tetK* and *tetM* although they were phenotypically tetracycline-resistant. *tetM* was detected in 37 isolates consisting of 30 ST239, six CC5-MRSA-VI + SCCfus and a CC5-MRSA-VI-paediatric clone. *tetK* was detected in 17 isolates with different genetic backgrounds. Both *tetK* and *tetM* occurred together in two ST239 isolates.

The 13 chloramphenicol-resistant isolates harboured *cat* (chloramphenicol acetyl transferase) (N = 7) or *fexA* (chloramphenicol/florfenicol exporter) which confers resistance to chloramphenicol and florfenicol (N = 6). The *fexA* was detected in t688 isolates belonging to CC5.

All trimethoprim – resistant isolates were positive for *dfrS1* (dihydrofolate reductase gene). Similarly, all high-level mupirocin-resistant isolates were positive for *mupA* (isoleucyl-tRNA synthetase) that mediates high-level mupirocin resistance.

Microarray also detected resistance determinants to antibiotics that were not tested phenotypically. These included *fosB* that is associated with fosfomycin resistance, *sat* (streptothrin acetyltransferase) that is associated with strepthtothrin resistance, *Sdrm*, a putative transport protein whose function is unknown. The *qacA* and *qacC* (multidrug efflux proteins) that mediate resistance to quaternary ammonium compounds. *fosB* was detected in 73 of the 120 isolates making it one of the common resistance determinants in a variety of genetic backgrounds.

### Detection of virulence determinants

Microarray analysis revealed the presence of different virulence determinants including regulatory genes (accessory gene regulators (agr), toxins including, Panton-Valentine leucocidin (PVL), haemolysins, leucocidins, Staphylococcal enterotoxins (SE), toxic shock syndrome toxins TSST-1), epidermal cell differentiation inhibitors, capsular polysaccharides (cap), biofilm associated factors (*icaA/ C/D*) and immune evasion cluster. All isolates were positive for haemolysins genes, *hl, hla, hlb, hllll* and *hld*, biofilm-encoding genes, *icaA/C/D* and clumping factor A (*clfA*) and clumping factor B (*clfB*). The isolates belonged to four accessory gene regulator (agr) types; agr I to agr IV. The leading *agrI* carrying isolates (N = 64) belonged to CC6, CC8, CC22, CC45, CC59, CC97, CC152 and CC361. The agr II isolates belonged to CC5, CC15, ST772 and ST2867, while *agr* III isolates were of CC1, CC30, CC80 and CC88. A single CC121 isolate carried *agr IV*. CC1 isolates, except ST772-MRSA-V, CC6, ST239, CC15, CC30, CC45, CC59, CC80, CC88, CC121 and CC361 were positive for capsular polysaccharide type 8 gene, *cap8*.The rest of the isolates carried *cap5*.

Genes for PVL was detected in 37 (30.8%) of the 120 isolates that belonged to CC1 (N = 9), CC5 (N = 4), CC22 (N = 5), CC30 (N = 7), CC59 (N = 1), CC80 (N = 7), CC88 (N = 1), and CC152 (N = 2).

One hundred and three (85.8%) of the 120 isolates harboured at least one Staphylococcal enterotoxins (SE) gene. The most prevalent SE genes were *egc* (N = 46), *sea* (N = 45), *seg* (N = 45), *sei* (N = 43), *sek* (N = 39), *seq* (N = 39), *seb* (N = 32), *sej* (N = 15), *sed* (N = 14) and *seh* (N = 8). Both *sec* and *sel* were detected in four isolates each. Most of the 103 isolates harboured two or more SE genes. Only five isolates carried single SE genes. Thirty-eight isolates carried two SE genes, 26 isolates carried three SE genes with the commonest combination being *seg*, *sei* and *egc* found in 18 of the isolates. Four SE genes were detected in 15 isolates and 19 isolates carried five or six SE genes. Four isolates consisting of three ST772-MRSA-V and one CC5-MRSA-V + PVL harboured six SE genes (*sea, sed, seg, sei, sej* and *egc*). Eighteen isolates belonging to CC15 (N = 3) and CC97 (N = 4), CC80 (N = 7), CC152 (N = 1), CC2250/2277 (N = 1) and ST2867 (N = 2) did not harbour SE genes. The genes for toxic shock syndrome toxin, *tst*, was detected in six isolates belonging to CC22-MRSA-IV[tst + ] (N = 3), CC30-MRSA-[VI + fus] +PVL (N = 2) and CC5-MRSA-IV[Paediatric clone] (N = 1). Eight isolates belonging to CC80 were positive for *etD* that codes for exfoliative toxin D.

Genes for epidermal cell differentiation inhibitors type A, *edinA*, was detected in two isolates belonging to CC5-IV-MRSA, and *edinB*, that codes for epidermal cell differentiation inhibitor type B, was detected in eight CC80-MRSA-IV, two CC152-MRSA-V + fus, and two ST2867-MRSA-V isolates.

The MRSA isolates varied in the carriage of genes for the immune evasion clusters (IEC), *sak* (staphylokinase), *chp* (chemotaxis inhibiting protein) and *scn* (staphylococcal complement inhibitor). Fifty-five (45.8%) of the 120 isolates carried all three IEC determinants, *sak. chp/scn*. These included 28 of the 30 ST239 – MRSA-III isolates, CC5-MRSA-IV, WA-MRSA-121, CC5-MRSA-IV (Paediatric clone), CC5-MRSA-V, WA MRSA-11/34/35/90/108, CC22-IV-MRSA, CC30-MRSA-IV, CC45-MRSA-[VI + fus] and CC88-MRSA-IV. Forty-six isolates carried *sak* and *Scn* while isolates of CC59 and CC5-MRSA-VI, new paediatric clone harboured chp and *sak*. The CC15-MRSA-V isolates harboured *chp* and *scn*. ST772-MRSA-V isolates harboured only *scn* while an ST239-MRSA-III + ccrC isolate lacked any of the IEC genes.

## Discussion

Due to constant changes in the epidemiology of MRSA strains it is necessary to conduct regular surveillance to determine clonal MRSA populations circulating in healthcare facilities to understand the evolution and dissemination of specific MRSA clones causing infections for the application of suitable infection control and preventive measures. The results of the present study have provided updates on the distribution and types of MRSA clones that circulated in the hospital in 2016. The isolates belonged to diverse genetic backgrounds with 60.3% and 39.7% of the MRSA isolates carrying the CA-MRSA and HA-MRSA genotypes respectively which is consistent with global trends^5,6^, and with recent reports that CA-MRSA are increasing in Kuwait hospitals^20,21^. The MRSA isolates belonged to six SCC*mec* types, 56 *spa* types and 16 clonal complexes (CC), with CC8, CC5, CC1, CC22, CC6, CC80, CC30 and CC97 as the common CCs highlighting the significant changes in the clonal composition of MRSA in the hospital since the last study in 1996–2001^16^. The reasons for the proliferation of the diverse MRSA clones observed in this study is not fully clear. However, the admission of an increasing number of patients already colonized with community genotypes of MRSA into the hospital may offer partial explanation. It is also worth noting that this observation is consistent with observations in different centres where CA-MRSA clones are overtaking the traditional HA-MRSA clones^5,6,14^.

The study showed that 30 (25%) of the 120 isolates analysed by DNA microarray were ST239-MRSA-III making ST239-MRSA-III the dominant MRSA clone in the hospital in 2016 as was the case in 1996–2001^16^. However, whereas MRSA isolates obtained in 1996–2001 belonged to a single dominant clone, ST239-MRSA-III^18^, those obtained in 2016 belonged to diverse genetic backgrounds. Significantly, although ST239-MRSA-III was the dominant genotype in this study as was the case in 1996–2001^16^, and 2005–2007^19^, their *spa* types were different. Whereas in 1996–2001, the dominant *spa* types were t037 and t421, the *spa* types of the 2005–2007 isolates were t421 and t945^19^, and those isolated in 2016 were dominated by t860 and t945.The introduction of t860 and its replacement of t037 and t421 subtypes demonstrates the constant evolution of the ST239-MRSA–III clone which may explain its successful persistence in the hospital. Similarly, ST239-III-MRSA was the common HA-MRSA clone in Saudi Arabia^22,23^, Iran^24^, and China^25^. In contrast, CA-MRSA clones are the dominant MRSA clones among recent MRSA isolates in other hospitals in Kuwait^18,20,21^.

The PVL-positive CC22-IV-MRSA, comprising t852, t005, and t11836, was the most prevalent CC22-MRSA-IV variant followed by the Middle Eastern variant in this study. In contrast, the *tst*-positive CC22-MRSA-IV, also known as the UK-EMRSA-15/Middle Eastern variant, was the most common variant in the other hospitals in Kuwait^21,26^. It is uncertain whether this observation signals a shift in the distribution of CC22-MRSA-IV variants in Kuwait. However, CC22-MRSA-IV [PVL^+]^/t852 was also reported as the dominant CC22-MRSA-IV variant in Qatar^27^.

The CC97 (ST97-MRSA-V) isolates were first reported in this hospital in 2007 as a cause of an outbreak in a neonatal intensive care and special care units^28^. However, until now, no further CC97-MRSA-V isolates were detected in the hospital following the termination of the outbreak. Curiously, the current CC97-MRSA-V isolates, except one (t359), belonged to *spa* t267, and were resistant to gentamicin, kanamycin, and fusidic acid similar to the resistance profile of the outbreak isolates even though they were isolated many years apart. Unfortunately, the *spa* type for the outbreak strains were not determined which makes it difficult to establish whether the same clone reappeared in the hospital in 2016. However, the *spa* t267 of the current isolates is different from *spa* t2297 and t1234 that were reported previously among CC97-MRSA-V in other hospitals in Kuwait^18,20^.

We noticed an isolate belonging to CC59 for the first time in Kuwait in this study. CC59 is a predominant CA-MRSA clone in Taiwan^29^, but has also been isolated in Hong Kong^30^, Japan^31^, and Western Australia^31^. However, it is rare in the Arabian gulf region. Hence, this report of CC59 in Kuwait suggests its recent importation into the country. The isolate in this report shares characteristics with the Taiwan (ST59/952-V_T_ /PVL) and Western Australian (ST59-IV) WA-MRSA-118 clones suggesting a common origin.

We observed that the axilla, groins and nose were colonised mostly by the HA-MRSA clone, ST239-MRSA-III, whereas the other sites were dominated by heterogeneous CA-MRSA clones. This is consistent with previous observations that the groin, axilla and nose are often colonised by ST239-MRSA-III strains which usually serve as sources of MRSA transmission^1,5,14^. Also, the dominance of CA-MRSA isolates in wound, pus and skin samples is consistent with earlier reports that CA-MRSA cause predominantly skin and soft tissue infections^1,4,5,8,14^. Furthermore, the isolation of CA-MRSA isolates from blood, and respiratory samples confirms their capacity to cause different types of infections other than skin and soft tissue infections^1,6,14^.

A high proportion of the isolates were resistant to multiple antibiotics including fusidic acid (63.4%), gentamicin (54.5%), tetracycline (54.5%), erythromycin (52.2%), ciprofloxacin (49.8%) and trimethoprim (21.5%). High prevalence of fusidic acid resistance is common among MRSA obtained in Kuwait hospitals. In recent studies, fusidic acid resistance was detected in 16.5% of MRSA obtained in the Maternity hospital^21^, and in 41.2% of MRSA obtained in Kuwait hospitals between 2011 and 2015^32^. The higher prevalence reported in the present study highlights an increasing trend for fusidic acid resistance among MRSA isolated in Kuwait hospitals.The high prevalence of fusidic acid resistance among MRSA isolates was attributed in part to the emerging chimeric genetic elements comprising SCC*mec* elements and the fusidic acid resistance determinant, *fusC* (SCCfusC)^20^ as also seen in this study (Table 1). The chimeric genetic element possibly facilitates the transmission of fusidic acid resistance in isolates belonging to different genetic backgrounds. The increasing prevalence of resistance to gentamicin, tetracycline, erythromycin, ciprofloxacin seen in this study have also been previously reported in MRSA otained from hospitals in Kuwait^16,18–20^.

We detected genes for multiple virulence factors including adhesins, haemolysins, enterotoxins and immune evasion clusters. However, the isolates differed in the prevalence of genes for PVL, enerotoxins, agr and capsular polysacharide (cap).The isolates belonged to cap5 (38.3%) and cap8 (61.7%) in agreement with previous observations that most clinical *S. aureus* isolates harbour *cap5* or *cap8*^33^. The higher prevalence of *cap8* in this study mirrors the results of a previous study that showed that 57% *S. aureus* obtained from food handlers in Kuwait restaurants carried *cap8*^34^. Similarly, *cap8* was prominent among *S.aureus* obtained from patients in Gabon^35^. This study also revealed that cap type was associated with defined genetic backgrounds.

The agrI was the leading agr type among the isolates in this study followed by agrII and agrIII. Similarly, most (50%) of *S. aureus* from food handlers in Kuwait restaurants belonged to agrI^34^. AgrI was also the dominant agr type among MRSA isolates obtained in Egypt^36^, India^37^, Iran^38^, Nigeria^39^, Turkey^40^, and China^41^. The study further revealed that the agr types were linked to specific clonal complexes as have been observed in *S. aureus* isolated from human and food sources in China^41^.

We identified genes coding for PVL in 30.8% (37/120) of the isolates belonging to different genetic backgrounds. This was higher than the 14.6% prevalence of genes for PVL in *S.aureus* in the same hospital in 2009–2010^17^. The higher prevalence of PVL- gene- positive isolates in this study may reflect the diversity of current clones in the hospital.

We found genes for staphylococcal enterotoxins (SE) in 85.1% of the isolates with most of the isolates harbouring two to six SE genes.Whereas the prominent SE genes in our study were egc (38.3%), *sea* (37.5%), *seg* (37.5%), *sei* (35.8%), *sek* (32.5%) and *seq* (32.5%), *sea* was the most common SE gene detected in MRSA from patients in Turkey^40^, Iran^38^, and Nigeria^39^. Since the carriage of SE genes as well as genes for agr and cap are genotype specific, the dominance of these factors may reflect the dominance of specific genotypes in the different geographical regions.

In conclusion, the evidence gathered from typing our MRSA isolates have revealed the expansion of the population of MRSA clones over time with the healthcare-associated MRSA, ST239-MRSA-III, as the dominant clone despite the emergence of diverse clones with CA-MRSA genotypes. These findings warrant a review of infection control and prevention procedures for the application of effective control measures that will limit antibiotic usage leading to improved patients’ care.

## Materials and Methods

### Bacterial isolates

A total of 209 single patient MRSA isolates were obtained in the Microbiology laboratory of Farwaniya Hospital during a period of 12 months, from January 1 2016, to 31 December 2016. The isolates were collected from cultures of various clinical specimens as part of routine diagnostic care and submitted for molecular typing at the MRSA Reference Laboratory located at the department of Microbiology, Faculty of Medicine, Kuwait University. Only a single isolate from a patient is submitted for typing. Repeat isolates from the same patient are excluded. The isolates were obtained from wound (N = 52; 24.9%), blood (N = 25; 12%), pus (N = 22; 10.5%), Nasal (N = 6;2.8%), Groins (N = 7;3.3%), axilla (N = 5;2.4%), sputum (N = 5; 2.4%), eye (N = 4;1.9%), ear (N = 3;1.4%), fluids (N = 3;1.4%), high vaginal swabs (N = 2; 0.9%) and an unidentified swab (N = 1; 0.4%). All isolates were identified as *S. aureus* by their cultural characteristics including growth on blood agar (5% sheep blood), growth on mannitol salt agar, Gram stain, positive tube coagulase test and DNase tests. The isolates were stored at −80 °C in 40% glycerol broth (v/v in brain heart infusion broth) and sub cultured onto brain heart infusion agar for purity prior to investigations.

### Antimicrobial susceptibility testing

Antibiotic susceptibility testing was performed by the disk diffusion method according to the guidelines of the Clinical Laboratory Standards Institute (CLSI)^42^, with the following antimicrobial disks (Oxoid): benzyl penicillin (10U), cefoxitin (30 μg), amikacin (30 μg), kanamycin (30 μg), tobramycin (10 μg), mupirocin (200 μg and 5 μg), gentamicin (10 μg), erythromycin (15 μg), clindamycin (2 μg), Spectinomycin (25 μg), chloramphenicol (30 μg), tetracycline (10 μg), trimethoprim (2.5 μg), fusidic acid (10 μg), rifampicin (5 μg), ciprofloxacin (5 μg), teicoplanin (30 μg), and linezolid (30 μg). Minimum inhibitory concentration (MIC) for cefoxitin, mupirocin, vancomycin and teicoplanin were determined with Etest strips (AB BioMerieux, Marcy l′Etoile, France) according to the manufacturer’s instructions. The interpretation of the MIC values was based on the antibiotic breakpoint concentration recommended by the CLSI^42^. Susceptibility to fusidic acid was interpreted according to the British Society to Antimicrobial Chemotherapy^43^. *S. aureus* strains ATCC25923 and ATCC29213 were used as a quality control strain for disk diffusion and MIC determination respectively.

### SCC*mec* typing and spa typing

All MRSA isolates were characterized using SCC*mec* typing and spa typing. SCC*mec* typing was performed as described by Zhang *et al*.^10^, *Spa* typing was performed as described by Harmsen *et al*.^13^.

### DNA microarray

The *S. aureus* genotyping Kit 2.0 (Alere Technology, Jena, Germany) was used following protocols provided by the manufacturer. The method detects 334 target sequences including genes encoding species markers, PVL, SCC*mec*, capsule (*cap*), and accessory gene regulator (*agr*) group, enterotoxins, adhesins, and antibiotic resistance^4^. DNA microarray was used to assign the MRSA isolates to clonal complexes (CC) as described previously^11^.

## Supplementary information

Supplementary Table 1

Supplementary Table 2
