# Supplementary material for: Molecular Characterization of Methicillin- Resistant Staphylococcus aureus in a Tertiary Care hospital in Kuwait
Source: Sci Rep. 2019 Dec 6;9:18527. doi: 10.1038/s41598-019-54794-8 (PMC6898362; doi:10.1038/s41598-019-54794-8)
Supplement: Supplementary file 1 — Supplementary Table 1 [file 41598_2019_54794_MOESM1_ESM.pdf]

Supplementary Table 1.

Molecular Characterization of Methicillin- Resistant *Staphylococcus aureus* in a Tertiary Care hospital in Kuwait.

Wadha Alfouzan,[1,2] Edet E. Udo,[2] Azizah Modhaffer,[1] Asma'a Alosaimi,[1]

1. Microbiology Unit, Department of Laboratory Medicine, Farwaniya hospital, Ministry of Health, Kuwait.

2. Department of Microbiology, Faculty of Medicine, Kuwait University, Jabriya, Kuwait.

Running title: MRSA in a tertiary care hospital in Kuwait.

Key words: MRSA, virulence factors, Antibiotic resistance, Molecular typing, DNA microarray

Correspondence to

Edet E Udo PhD

Department of Microbiology

Faculty of Medicine, Kuwait University

P. O. Box 24923

Safat, 13110. KUWAIT

EDET@hsc.edu.kw

+965 24636773

Supplementary Table

Serial No.	Vial No.	Specimen	Penicillin	Gentamicin	Kanamycin	Erythromycin	Clindamycin	Chloramphenicol	Tetracycline	Trimethoprim	Fusidic Acid	Ciprofloxacin	Mupirocin 200	Vancomycin mg\L	Teicoplanin mg\L	SCC MEC	Spa Type
1	17256	Axilla	R	R	R	R	C	S	R	S	R	R	LR	1	1	III	t945
2	4359	Axilla	R	R	R	R	C	S	R	S	R	R	LR	1.5	1.5	III	t860
3	4783	Axilla	R	R	R	R	C	S	R	S	R	R	LR	2	2	III	t860
4	4814	Axilla	R	R	R	R	C	S	R	S	R	R	LR	2	2	III	t860
5	4918	Axilla	R	R	R	R	C	S	R	S	R	R	LR	2	1.5	III	t860
6	5281	Axilla	R	R	R	R	C	S	R	S	R	R	LR	1.5	2	III	t860
7	5372	Axilla	R	R	R	R	C	S	R	S	R	R	LR	1	1.5	III	t945
8	5734	Axilla	R	R	R	R	C	S	R	S	R	R	LR	0.75	1.5	III	t945
9	17373	Axilla	R	R	R	R	C	S	R	S	R	R	LR	4	2	III	t860
10	17456	Axilla	R	R	R	R	C	S	R	S	R	R	LR	1	1	III	t860
11	18082	Axilla	R	R	R	R	C	S	R	S	R	R	LR	2	3	III	t945
12	18164	Axilla	R	R	R	R	C	S	R	S	R	R	LR	1.5	1.5	III	t860
13	18509	Axilla	R	R	R	R	C	S	R	S	R	R	LR	1	1	III	t860
14	17336	Blood	S	S	S	S	S	S	R	R	R	S	S	1	1.5	V	t535
15	4466	Blood	R	R	R	R	C	S	R	S	R	R	LR	1.5	2	III	t860
16	4590	Blood	S	S	S	R	C	R	R	S	S	R	S	1	1.5	V	t688
17	4591	Blood	S	S	S	R	C	R	R	S	S	R	S	1	2	V	t688
18	4816	Blood	S	R	R	S	S	S	S	S	R	S	S	1	1	V	t127
19	4881	Blood	R	S	R	S	S	S	R	S	R	S	S	1	1	IV	t044
20	5222	Blood	R	R	R	S	S	S	S	S	R	R	S	2	3	IV	t267
21	5284	Blood	R	S	S	S	S	S	R	S	R	S	S	1	1.5	V	t2207
22	5503	Blood	R	R	R	R	C	S	R	S	R	R	LR	1.5	2	VI	t860
23	5581	Blood	R	R	R	S	S	S	S	S	R	S	S	0.75	1	V	t267
24	5603	Blood	R	R	R	R	I	S	S	R	S	R	S	0.75	1	IV	t852
25	5795	Blood	R	R	R	S	S	S	S	S	S	S	S	2	2	V	t306
26	5910	Blood	R	R	R	S	S	S	R	S	R	R	S	1	1	IV	t127
27	5943	Blood	R	S	S	S	S	R	R	R	R	S	S	2	2	V	t688
28	17377	Blood	S	S	S	S	S	S	S	S	S	S	S	4	3	IV	t6845
29	17622	Blood	R	S	S	S	S	S	S	S	S	S	S	1	1.5	IV	t8168
30	17681	Blood	R	S	S	S	S	R	R	R	R	S	S	1.5	1.5	VI	t535
31	17728	Blood	R	S	S	S	S	S	S	S	S	S	S	1.5	1.5	V	t148
32	17746	Blood	R	S	S	S	S	S	S	R	S	S	S	1.5	1.5	IV	t701

Supplementary Table

Serial No.	Vial No.	Specimen	Penicillin	Gentamicin	Kanamycin	Erythromycin	Clindamycin	Chloramphenicol	Tetracycline	Trimethoprim	Fusidic Acid	Ciprofloxacin	Mupirocin 200	Vancomycin mg\L	Teicoplanin mg\L	SCC MEC	Spa Type
33	17806	Blood	R	S	S	S	S	S	S	S	S	S	S	1	0.75	IV	t790
34	18057	Blood	R	S	S	S	S	S	S	S	S	S	S	1.5	1	IV	t019
35	18231	Blood	R	S	S	R	I	S	S	S	S	R	S	1	0.25	IV	t032
36	18347	Blood	R	S	S	R	I	S	S	R	R	R	S	1.5	2	V	t311
37	18455	Blood	R	R	R	S	S	S	R	S	R	S	S	1.5	1.5	V	t267
38	18458	Blood	R	S	S	R	I	R	R	S	S	S	S	2	2	V	t688
39	4810	Ear	R	S	S	S	S	R	R	S	R	S	S	1	1	VI	t688
40	4812	Ear	R	S	S	R	I	S	S	R	R	R	S	1	1.5	III	t311
41	4851	Ear	S	R	R	S	S	S	S	S	S	S	S	1	1	IV	t019
42	5587	Ear	R	R	R	R	C	S	R	S	R	R	LR	1.5	2	III	t860
43	17389	Ear	R	S	S	R	S	S	S	S	S	S	S	0.75	0.75	IV	t008
44	4438	Eye	R	S	S	S	S	R	R	R	R	R	S	0.75	2	IV	t688
45	4779	Eye	S	S	S	R	C	S	R	S	S	S	S	1.5	1.5	V	t311
46	4916	Eye	R	R	R	R	I	S	S	S	R	S	S	1	2	V	t267
47	4984	Eye	R	S	S	S	S	S	S	R	S	S	S	1	1.5	IV	t223
48	17928	Eye	R	S	S	R	C	S	S	S	R	R	S	2	1.5	V	t311
49	5037	Fluid	R	S	R	R	S	S	S	R	S	R	S	2	0.75	IV	t363
50	5243	Fluid	R	S	S	R	I	S	S	S	S	S	S	1	1.5	IV	t002
51	17291	Groin	R	R	R	R	C	S	R	S	R	R	LR	2	1	III	t860
52	4520	Groin	R	R	R	R	C	S	R	S	R	R	LR	1.5	1.5	III	t945
53	4647	Groin	R	R	R	R	C	S	R	R	R	R	LR	1.5	2	III	t860
54	4781	Groin	R	R	R	R	C	S	R	S	R	R	LR	2	2	III	t860
55	4782	Groin	R	R	R	R	C	S	R	S	R	R	LR	2	2	III	t860
56	5260	Groin	R	R	R	R	C	S	R	S	R	R	LR	1.5	1.5	III	t860
57	5474	Groin	R	R	R	R	C	S	R	S	R	R	LR	1	1.5	III	t860
58	5794	Groin	R	R	S	S	S	S	R	S	R	R	LR	2	2	V	t860
59	5797	Groin	R	R	R	S	S	S	R	S	R	R	LR	2	2	III	t945
60	5830	Groin	R	R	R	R	C	S	R	R	R	S	S	1.5	1	III	t860
61	5942	Groin	R	R	R	R	C	R	R	S	R	R	S	1.5	2	V	t037
62	17569	Groin	R	R	R	R	C	S	R	S	R	R	LR	2	2	III	t860
63	18081	Groin	R	R	R	R	C	S	S	S	R	R	LR	1.5	1.5	III	t860
64	18109	Groin	R	R	R	R	C	S	R	S	R	R	LR	2	1.5	III	t945

Supplementary Table

Serial No.	Vial No.	Specimen	Penicillin	Gentamicin	Kanamycin	Erythromycin	Clindamycin	Chloramphenicol	Tetracycline	Trimethoprim	Fusidic Acid	Ciprofloxacin	Mupirocin 200	Vancomycin mg\L	Teicoplanin mg\L	SCC MEC	Spa Type
65	18110	Groin	R	R	R	R	C	S	R	S	R	R	LR	2	1.5	III	t860
66	18186	Groin	R	R	R	R	C	S	R	S	R	R	LR	2	1.5	III	t860
67	18345	Groin	R	R	R	R	C	S	S	S	R	S	LR	1.5	1.5	III	t945
68	18424	Groin	R	R	R	R	C	S	R	S	R	R	LR	1.5	1.5	III	t860
69	4919	HVS	R	S	S	S	S	S	S	S	S	S	S	1.5	1.5	IV	t304
70	4939	HVS	R	S	S	S	S	S	S	S	R	S	S	2	1	IV	t362
71	5087	HVS	R	S	R	S	S	S	R	S	R	S	S	1	0.75	IV	t044
72	5219	HVS	R	R	R	S	S	S	R	S	R	R	S	2	2	V	t044
73	5777	HVS	R	S	S	S	S	R	S	R	R	S	S	1.5	2	V	t688
74	4439	Nasal	R	R	R	R	C	S	R	R	R	R	LR	1.5	2	III	t860
75	4780	Nasal	R	R	R	R	C	S	R	S	R	R	LR	3	2	III	t860
76	4982	Nasal	R	R	R	R	C	S	R	R	R	R	LR	2	1.5	III	t860
77	4983	Nasal	R	R	R	R	C	S	R	S	R	R	LR	1.5	1	III	t860
78	17391	Nasal	R	R	R	R	C	S	R	S	R	R	HLR	1	1.5	III	t860
79	17457	Nasal	R	R	R	R	C	S	R	S	R	R	LR	1.5	2	III	t860
80	17600	Nasal	R	S	S	S	S	S	S	S	S	S	S	2	2	IV	t304
81	17724	Nasal	R	R	R	R	C	S	R	S	R	R	LR	1.5	1.5	III	t713
82	17726	Nasal	R	R	R	R	C	S	R	S	R	R	LR	1	1.5	III	t860
83	17730	Nasal	R	R	R	R	C	S	R	S	R	R	LR	1.5	2	III	t860
84	18163	Nasal	R	R	R	R	C	S	R	S	R	R	LR	2	2	III	t860
85	18208	Nasal	R	R	R	R	C	S	R	S	R	R	LR	2	1.5	III	t860
86	18387	Nasal	R	R	R	R	C	S	R	S	R	S	LR	1.5	1.5	III	t945
87	18510	Nasal	S	S	S	S	S	R	S	S	R	S	S	1	0.75	IV	t14700
88	4777	Others	R	S	S	S	S	S	S	R	S	S	S	1.5	2	IV	t019
89	4880	Others	R	R	R	S	S	S	R	S	R	R	S	1.5	2	V	t127
90	4923	Others	R	R	R	S	S	S	S	S	S	S	S	1	1.5	V	t003
91	5283	Others	R	S	S	R	I	S	S	S	S	R	S	2	1	IV	t304
92	5375	Others	R	S	R	S	S	S	R	S	S	S	S	0.75	1.5	IV	t044
93	5717	Others	R	R	R	R	I	S	R	R	R	R	HLR	0.75	1.5	III	t425
94	5876	Others	S	S	S	R	S	S	S	R	S	S	S	2	2	V	t743
95	17491	Others	R	R	R	R	C	S	R	S	R	R	LR	2	2	III	t860
96	17721	Others	R	S	S	S	S	R	R	R	R	S	S	1.5	1.5	VI	t688

Supplementary Table

Serial No.	Vial No.	Specimen	Penicillin	Gentamicin	Kanamycin	Erythromycin	Clindamycin	Chloramphenicol	Tetracycline	Trimethoprim	Fusidic Acid	Ciprofloxacin	Mupirocin 200	Vancomycin mg\L	Teicoplanin mg\L	SCC MEC	Spa Type
97	17745	Others	R	S	S	S	S	S	S	S	S	S	S	1.5	1.5	IV	t002
98	18283	Others	R	R	R	S	S	S	S	R	S	R	S	1.5	1.5	IV	t852
99	18456	Others	R	R	R	R	C	S	R	S	R	R	LR	2	2	III	t860
100	18507	Others	R	S	S	R	I	S	S	S	S	S	S	1.5	1.5	IV	t304
101	18511	Others	R	R	R	R	I	S	S	R	S	R	S	0.5	0.75	IV	t005
102	18525	Others	S	R	R	S	S	S	R	S	R	S	S	0.5	1	V	t16302
103	4369	Pus	R	R	R	S	S	S	S	R	S	S	S	1	1.5	V	t657
104	4734	Pus	R	R	R	S	S	S	R	S	R	S	S	1	1	V	t127
105	4808	Pus	R	S	R	S	S	S	R	S	R	S	S	1.5	1	IV	t044
106	4854	Pus	R	S	S	R	I	R	R	S	S	S	S	1	1.5	V	t688
107	4914	Pus	S	R	R	S	S	S	R	S	R	S	S	1.5	1.5	V	t127
108	4920	Pus	R	S	S	S	S	S	S	S	S	S	S	1	2	IV	t019
109	4922	Pus	R	S	S	S	S	S	S	S	R	S	S	2	2	IV	t044
110	5145	Pus	R	S	S	S	S	S	S	S	R	S	S	1.5	2	I	t018
111	5161	Pus	R	S	R	S	S	S	S	S	R	S	S	1	1.5	IV	t044
112	5214	Pus	R	R	R	R	S	S	S	S	R	R	S	3	3	IV	t127
113	5350	Pus	R	S	R	R	I	S	S	S	S	R	S	1	1	IV	t16186
114	5353	Pus	R	S	S	S	S	S	S	S	S	S	S	1	1.5	IV	t304
115	5430	Pus	R	R	R	S	S	S	S	R	S	S	S	0.75	1	IV	t005
116	5652	Pus	R	R	R	R	I	S	S	S	R	R	S	1	1.5	IV	t127
117	5655	Pus	R	S	S	R	I	S	S	S	S	R	S	1	1	V	t002
118	5716	Pus	S	S	R	S	S	S	S	S	S	S	S	1	1.5	IV	t042
119	17390	Pus	R	S	S	S	S	S	S	S	S	S	S	1.5	1.5	IV	t019
120	17393	Pus	R	S	S	S	S	S	S	S	R	S	S	0.75	1	IV	t002
121	17424	Pus	R	S	S	R	I	S	S	S	S	S	S	1.5	2	IV	t304
122	17425	Pus	R	R	R	R	C	S	R	S	R	S	S	1.5	2	V	t127
123	17624	Pus	R	R	R	S	S	S	S	R	S	S	S	1	0.75	IV	t005
124	17723	Pus	R	R	R	R	S	S	S	R	S	R	S	1	2	V	t5414
125	4347	Skin	R	R	R	S	S	S	S	S	R	S	S	1.5	2	V	t11206
126	4811	Skin	R	S	S	R	I	S	S	R	R	R	S	1.5	2	III	t311
127	5244	Skin	R	S	S	R	I	S	S	R	S	S	S	0.5	0.38	IV	t223
128	5326	Skin	S	R	R	R	I	S	R	S	R	S	S	1	0.75	V	t127

Supplementary Table

Serial No.	Vial No.	Specimen	Penicillin	Gentamicin	Kanamycin	Erythromycin	Clindamycin	Chloramphenicol	Tetracycline	Trimethoprim	Fusidic Acid	Ciprofloxacin	Mupirocin 200	Vancomycin mg\L	Teicoplanin mg\L	SCC MEC	Spa Type
129	17374	Skin	R	S	S	S	S	S	S	R	S	S	S	2	3	IV	t16202
130	17725	Skin	R	R	R	R	C	S	R	S	R	R	LR	2	1.5	III	t860
131	17729	Skin	R	R	R	R	C	S	R	S	R	R	LR	2	2	III	t860
132	4852	Sputum	R	S	S	R	I	S	S	S	S	S	S	2	1.5	IV	t002
133	5163	Sputum	R	S	S	R	I	S	S	S	S	S	S	0.75	1	IV	t002
134	5911	Sputum	R	S	S	R	I	S	R	S	S	S	S	1.5	2	IV	t002
135	17519	Sputum	R	R	R	R	C	S	R	S	R	R	LR	1	2	III	t860
136	17988	Sputum	R	S	S	R	I	S	S	R	R	R	S	2	2	V	t311
137	17517	Swab	R	R	R	R	C	S	R	S	R	R	LR	1	2	III	t860
138	18350	Throat	R	R	R	R	C	S	R	S	R	R	LR	1	0.75	III	t945
139	4522	Tissue	R	S	S	S	S	S	S	S	S	S	S	1.5	2	IV	t304
140	4371	Tracheal	S	R	R	R	C	S	S	S	S	R	S	1	2	II	t045
141	4413	Tracheal	R	S	S	R	I	S	S	R	R	R	S	1	2	V	t311
142	4853	Tracheal	R	R	R	R	C	S	R	R	R	R	LR	1	1.5	III	t860
143	4924	Tracheal	R	S	S	R	C	S	S	S	S	S	S	1	1.5	I	t018
144	5604	Tracheal	R	S	S	S	S	S	S	R	S	S	S	0.5	1	IV	t105
145	5718	Tracheal	R	S	S	S	S	R	R	R	R	R	S	1.5	1	IV	t688
146	17516	Tracheal	S	S	S	R	C	S	S	S	S	R	S	1	2	IV	t304
147	17518	Tracheal	R	S	S	S	S	S	R	S	S	S	S	1	1	IV	ND
148	17623	Tracheal	R	S	S	R	I	S	R	S	S	S	S	0.75	1	IV	t690
149	17771	Tracheal	R	R	R	S	S	S	S	S	R	S	S	1	1.5	V	t359
150	17906	Tracheal	R	S	S	S	S	S	S	S	S	S	S	1.5	2	IV	t304
151	17935	Tracheal	R	R	R	R	C	S	R	S	R	R	LR	1	1.5	III	t860
152	18080	Tracheal	R	S	S	S	S	R	R	S	R	S	S	2	1.5	VI	t688
153	18385	Tracheal	R	R	R	R	C	S	R	S	R	S	HLR	2	2	III	t860
154	18386	Tracheal	R	S	S	S	S	S	S	S	S	S	S	1.5	1.5	IV	t304
155	5657	Unknown	R	S	S	S	S	S	S	S	S	S	S	1.5	1	IV	t1752
156	5829	Urine	R	S	S	S	S	S	S	S	S	S	S	1	1.5	V	t148
157	17727	Urine	R	R	R	R	S	S	S	S	R	S	S	1.5	1.5	V	t127
158	4467	Wound	R	R	R	R	C	S	R	S	R	R	LR	0.75	1.5	III	t945
159	4524	Wound	R	S	S	S	S	S	S	S	S	S	S	1.5	2	IV	t019
160	4645	Wound	R	S	S	S	S	S	S	R	S	S	S	1.5	2	IV	t008

Supplementary Table

Serial No.	Vial No.	Specimen	Penicillin	Gentamicin	Kanamycin	Erythromycin	Clindamycin	Chloramphenicol	Tetracycline	Trimethoprim	Fusidic Acid	Ciprofloxacin	Mupirocin 200	Vancomycin mg\L	Teicoplanin mg\L	SCC MEC	Spa Type
161	4855	Wound	R	R	R	S	S	S	S	S	R	S	S	1	1	V	t127
162	4857	Wound	R	R	R	R	I	S	R	S	R	R	LR	1.5	0.75	III	t945
163	4912	Wound	R	S	S	R	I	R	R	S	S	S	S	1	1	V	t688
164	4979	Wound	R	R	R	R	C	S	R	S	R	R	LR	1.5	1	III	t945
165	5138	Wound	R	R	R	S	S	S	R	S	R	S	S	1.5	1.5	V	t267
166	5140	Wound	R	R	R	S	S	S	S	S	R	S	S	1.5	2	V	t127
167	5162	Wound	R	R	R	R	C	S	S	S	R	S	S	0.5	1	V	t127
168	5215	Wound	R	S	R	S	S	S	R	S	R	R	S	1.5	3	IV	t044
169	5323	Wound	R	S	S	S	S	S	S	R	S	R	S	1.5	1	V	t2393
170	5327	Wound	R	R	R	R	C	S	R	S	R	R	LR	1	1	III	t860
171	5328	Wound	R	R	R	R	C	S	R	S	R	R	LR	2	1	III	t945
172	5349	Wound	R	S	S	R	S	S	S	S	S	S	S	1.5	1.5	IV	t304
173	5373	Wound	R	R	R	R	C	S	R	S	R	R	LR	0.75	0.38	III	t945
174	5501	Wound	R	S	S	R	I	S	S	S	S	R	S	1.5	2	III	t304
175	5605	Wound	R	S	S	S	S	S	R	S	R	S	S	1	1	III	t355
176	5658	Wound	R	S	R	S	S	S	R	S	R	R	S	1	1.5	IV	t044
177	5662	Wound	R	S	S	S	S	R	R	R	R	S	S	1.5	1	V	t688
178	5712	Wound	R	R	R	S	S	S	S	S	R	S	S	1	1.5	IV	t267
179	5719	Wound	R	R	R	R	I	S	R	R	S	R	S	1	1.5	IV	t223
180	5827	Wound	R	S	S	R	I	S	S	S	S	S	S	1	1.5	IV	t304
181	5831	Wound	R	R	R	R	C	S	R	R	R	R	LR	1	1.5	III	t860
182	5853	Wound	R	S	S	R	C	R	R	R	S	S	S	1	1	V	t688
183	5854	Wound	S	S	R	S	S	S	S	S	S	S	S	0.5	0.5	IV	t044
184	5859	Wound	S	S	R	R	C	R	S	S	S	S	S	0.75	1	V	t437
185	5875	Wound	S	S	S	S	S	S	S	S	S	S	S	1	1.5	V	t003
186	5891	Wound	R	S	S	S	S	S	S	R	R	R	S	1	1	V	t16185
187	5941	Wound	S	S	R	S	S	S	S	S	R	S	S	0.75	1.5	IV	t042
188	17372	Wound	R	R	R	R	C	R	R	S	R	R	LR	2	2	III	t945
189	17375	Wound	R	R	R	S	S	S	S	S	R	S	S	2	1.5	IV	t044
190	17376	Wound	R	S	S	S	S	S	R	R	S	R	S	3	2	V	t1839
191	17378	Wound	R	R	R	S	S	S	R	S	R	R	S	3	4	IV	t376
192	17394	Wound	R	S	S	S	S	S	S	R	S	S	S	2	1.5	IV	ND

Supplementary Table

Serial No.	Vial No.	Specimen	Penicillin	Gentamicin	Kanamycin	Erythromycin	Clindamycin	Chloramphenicol	Tetracycline	Trimethoprim	Fusidic Acid	Ciprofloxacin	Mupirocin 200	Vancomycin mg\L	Teicoplanin mg\L	SCC MEC	Spa Type
193	17423	Wound	R	R	R	R	C	S	R	S	R	R	LR	2	2	III	t945
194	17426	Wound	R	R	R	S	S	S	R	S	R	S	S	3	2	V	t084
195	17720	Wound	R	S	R	R	C	S	S	S	S	S	S	1	1.5	IV	t002
196	17722	Wound	R	R	R	S	S	S	S	S	R	S	S	1.5	1.5	V	t314
197	17786	Wound	R	R	R	R	C	S	R	S	R	R	LR	1.5	1.5	III	t945
198	17872	Wound	R	R	R	R	C	S	R	S	R	R	LR	1	2	III	t860
199	17896	Wound	R	R	R	R	C	S	R	S	R	R	LR	1.5	1.5	III	t860
200	17907	Wound	R	S	S	S	S	S	S	S	S	S	S	1.5	2	IV	t12398
201	17908	Wound	R	S	R	S	S	S	R	S	R	S	S	1.5	2	IV	t044
202	18281	Wound	R	R	R	S	S	S	R	S	R	S	S	1.5	2	V	t084
203	18282	Wound	R	R	R	S	S	S	R	S	R	S	S	2	2	V	t084
204	18343	Wound	R	S	R	R	S	S	S	R	S	R	S	2	1.5	IV	t021
205	18344	Wound	R	R	R	S	S	S	S	R	S	S	S	1	1	IV	t11836
206	18457	Wound	R	R	R	R	C	S	R	S	R	R	LR	2	2	III	t860
207	18479	Wound	R	R	R	S	S	S	R	S	R	S	S	1.5	1.5	V	t127
208	18508	Wound	R	R	R	R	C	S	R	S	R	R	LR	1	1	III	t860
209	18512	Wound	R	S	R	S	S	S	S	R	S	R	S	1.5	1.5	IV	t3841
