# Supplementary material for: Molecular Characterization of Methicillin- Resistant Staphylococcus aureus in a Tertiary Care hospital in Kuwait
Source: Sci Rep. 2019 Dec 6;9:18527. doi: 10.1038/s41598-019-54794-8 (PMC6898362; doi:10.1038/s41598-019-54794-8)
Supplement: Supplementary file 2 — Supplementary Table 2 [file 41598_2019_54794_MOESM2_ESM.pdf]

Supplementary Table 2.

Molecular Characterization of Methicillin- Resistant *Staphylococcus aureus* in a Tertiary Care hospital in Kuwait.

Wadha Alfouzan,[1,2] Edet E. Udo,[2] Azizah Modhaffer,[1] Asma'a Alosaimi,[1]

1. Microbiology Unit, Department of Laboratory Medicine, Farwaniya hospital, Ministry of Health, Kuwait.

2. Department of Microbiology, Faculty of Medicine, Kuwait University, Jabriya, Kuwait.

Running title: MRSA in a tertiary care hospital in Kuwait.

Key words: MRSA, virulence factors, Antibiotic resistance, Molecular typing, DNA microarray

Correspondence to

Edet E Udo PhD

Department of Microbiology

Faculty of Medicine, Kuwait University

P. O. Box 24923

Safat, 13110. KUWAIT

EDET@hsc.edu.kw

+965 24636773

Supplementary Table 2. Characteristics of MRSA isolates characterized by microarray

CC	MRSA genotypes (N)	Spa types (N)	Antibiogram	Antibiotic resistance genes	Toxins	Miscellaneous
CC1	CC1-MRSA-V+SCCfus [PVL+] (5)	*t127 (4), ND (1)	Gm (4), K (4), E (1), CC (1), Cm (1), Te (5), Tp (1), FD (5), Cip (1)	*ermC, aacA-aphD, aphA3,cat, fusC, sat*	*PVL; sea, seh, sek, seq*	*argIII; cap8; sak/scn; icaA/C/D*
	CC1-MRSA-V+SCCfus (2)	**t127 (2)	Gm, K, E, FD	*msr(A), mphC, aacA-aphD, aphA3, sat, fusC, qacC*	*sea, seb, seh, sek, seq*	*argIII; cap8; sak/scn; icaA/C/D*
	CC1-MRSA-[V/V_T+fus] (PVL+) (1)	t127 (1)	Gm, K, E, CC, FD, Cm	*ermC, aacA-aphD,fusC, cat*	*PVL; sea, sed, seh, sek, seq*	*argIII; cap8; sak/scn; icaA/C/D*
	ST772-MRSA-V [PVL+], Bengal Bay Clone (3)	t657 (1), t1839 (1), t5414 (1)	Gm (2), K (2), E (1), Te (1), Tp (3), Cip (2)	*msr(A), mphC, aacA-aphD, aphA3, tetK, sat, fosB*	*PVL; sea, sec, seg, sei, sel, egc gene cluster*	*agrII; cap5; scn; icaA/C/D*
CC5	CC5-MRSA-IV, [PVL+/edinA+], WA MRSA-121 (1)	t105	Tp	*fosB*	*PVL; seg, sei, egc gene cluster*	*agrII; cap5; sak/chp/scn; icaA/C/D; edinA*
	CC5-MRSA-IV, [PVL+] (3)	***t002 (3)	E (2), CC (2), FD (1), HLR-Mup	*ermC, fusB, mupA, fosB*	*PVL; sea (N315), sed, seg, sei, sej, egc gene cluster*	*agrII; cap5; sak/scn; icaA/C/D*

CC	MRSA genotypes (N)	Spa types (N)	Antibiogram	Antibiotic resistance genes	Toxins	Miscellaneous
	CC5-MRSA-IV, Paediatric clone [tst1+] (1)	t002	K, E, CC,	*ermC, msrA, mphC, aphA3, sat, fosB*	*tst1; sec, seg, sei, sel, egc gene cluster*	*agrII; cap5; sak/chp/scn; icaA/C/D, edinA*
	CC5-MRSA-V [sed/j/r+], WA MRSA-11/34/35/90/108 (2)	****t306, *****t688	Gm (1), K (1), E (1), CC (1), Cm (1), Te (1),	*ermC, tetK, fexA, fosB, aacA-aphD*	*sea (N315), sed, seg, sei, sej, egc gene cluster*	*agrII; cap5; sak/scn; icaA/C/D*
	CC5-MRSA-V, WA MRSA-81/85/86/123 (1)	t311	E, CC, Te	*ermC, tetK, fosB*	*seg, sei, egc gene cluster*	*agrII; cap5; sak/chp/scn; icaA/C/D*
CC5	CC5-MRSA-V+SCCfus, WA MRSA-14/109 (5)	●t311 (4), ●●t16185 (1)	E (4), CC (4), Tp (4), FD (5), Cip (5)	*ermC, fusC, fosB*	*seb, seg, sei, egc gene cluster*	*agrII; cap5; sak; icaA/C/D*
	CC5-MRSA-VI, New Paediatric Clone (1)	t688	Cm, Te, FD	*dfrS1, tetM, fexA*	*sed, seg, sej, egc gene cluster*	*agrII; cap5; chp/scn; icaA/C/D*
CC5	CC5-MRSA-VI+SCCfus (6)	t535 (2), t688 (4)	Cm (5), Te (6), Tp (6), FD (6), Cip (2)	*dfrS1, fusC, tetM, fexA, fosB*	*sea (N315), sed, seg, sei, sej, egc gene cluster*	*agrII; cap5; sak/scn; icaA/C/D*

CC	MRSA genotypes (N)	Spa types (N)	Antibiogram	Antibiotic resistance genes	Toxins	Miscellaneous
	ST5/ST225-MRSA-II, Rhine-Hesse EMRSA/New York-Japan Clone (1)	t045	Gm, K, E, CC, Cip	*ermA, aadD, fosB*	*sea (N315), sed, seg, sei, sej, egc gene cluster*	*agrII; cap;5 sak/chp/scn; icaA/C/D*
CC6	CC6-MRSA-[IV+fus+ccrC] (1)	t14700 (1)	Cm, FD	*fusC, fosB*	*Sea*	*agrI; cap8; sak/scn; icaA/C/D*
	CC6-MRSA-IV, WA MRSA-51 (10)	t304 (6), t701 (1), t6845 (1), t8168 (1), ND (1)	E (3), CC (3), Te (1), Tp, (1), Cip (2)	*ermC, aadD, tetK, fosB*	*sea, seb*	*agrI; cap8; sak/scn; icaA/C/D*
CC8	CC8-MRSA-IV [sea-N315+] (1)	t008	Tp	*fosB*	*sea (N315), seb, sej, ser*	*agrI; cap5; sak/scn; icaA/C/D*
	CC8-MRSA-IV, UK-EMRSA-14/WA MRSA-5 (1)	t008	E	*msr(A), mphC, fosB*	*seb*	*agrI; cap5; sak/chp/scn; icaA/C/D*
	ST239-MRSA-III+ccrC, Vienna/Hungarian/Brazilian Clone (1)	t037	Gm, K, E, CC, Cm, Te, FD, Cip	*ermC, aacA-aphD, aphA3, sat, tetK, tetM, cat, fosB*	*sea, sek, seq*	*agrI; cap8; icaA/C/D*
	ST239-MRSA-III+SCCmer, Vienna/Hungarian/Brazilian Clone (28)	t713 (1), t860 (16), t945 (10), t1247 (1)	Gm (28), K (27), E (27), CC (27), Cm (1), Te (26), FD (28), Cip (26), HLR-Mup (2)	*ermA, aacA-aphD, aadD, aphA3, sat, mupA, cat, tetM, fosB, qacA*	*sek, seq*	*agrI ,cap8, Sak/chp/scn/ icaA/C/D*
	ST239-MRSA-III+SCCmer+ccrC, Vienna/Hungarian/Brazilian Clone (1)	t425	Gm, K, E, CC, Te, Tp, FD, Cip, HLR-Mup	*ermA, lnu(A), aacA-aphD, aphA3, aadD, sat, dfrS1, mupA, tetK, tetM, fosB*	*sea*	*agrI, cap8, ,icaA/C/D*

CC	MRSA genotypes (N)	Spa types (N)	Antibiogram	Antibiotic resistance genes	Toxins	Miscellaneous
CC15	CC15-MRSA-V+SCCfus (3)	t084 (2), t7583 (1)	Gm (3), K (3), Te (3), FD (3)	*lnu(A), aadD, aacA-aphD, fusC, tetK, fosB*	-	*agrII; cap8; chp/scn; icaA/C/D*
CC22	CC22-MRSA-IV [fnbB-,sec/l-], UK-EMRSA-15/Barnim EMRSA (1)	t032	E, CC, Cip	*dfrS1, ermC*	*seg, sei, egc gene cluster*	*agrI; cap5; sak/chp/scn; icaA/C/D*
	CC22-MRSA-IV [fnbB+], UK-EMRSA-15/Barnim EMRSA (2)	t223, t790	Gm (1), K (1), E (1), CC (1), Te (1), Tp (1)	*ermC, aacA-aphD, aadD, dfrS1, tet(K)*	*seg, sei, egc gene cluster*	*agrI; cap5; sak/chp/scn; icaA/C/D*
	CC22-MRSA-IV [PVL+] (5)	t005 (1), t852 (3), t11836 (1)	Gm (5), K (5), E (2), CC (2), Tp (4), Cip (1),	*ermC, aacA-aphD, aadD, dfrS1*	*PVL; seg, sei, egc gene cluster*	*agrI; cap5; sak/chp/scn; icaA/C/D*
	CC22-MRSA-IV [tst1+], UK-EMRSA-15/Middle Eastern variant (3)	t005, t223, t16202	Gm (1), K (1), E (2), CC (2), Tp (3)	*ermC, dfrS1*	*tst1; sea, seg, sei, egc gene cluster*	*agrI; cap5; sak/chp/scn; icaA/C/D*
CC30	CC30-MRSA-[VI+fus] (PVL+) (2)	t018 (2)	E (1), CC (1), FD (1)	*ermC, fusC, fosB*	*PVL; TST1; sea, seg, sei, egc gene cluster*	*agrIII; cap8; sak/chp/scn; icaA/C/D*
	CC30-MRSA-IV [PVL+], Southwest Pacific Clone (5)	t019 (3), t021 (1), t363 (1)	Gm (1), K (3), E (2), Tp (2), Cip (2)	*msr(A), mphC, aacA-aphD, aphA3, sat, fosB,*	*PVL; seg, sei, egc gene cluster*	*agrIII; cap8; sak/chp/scn; icaA/C/D*

CC	MRSA genotypes (N)	Spa types (N)	Antibiogram	Antibiotic resistance genes	Toxins	Miscellaneous
CC45	CC45-MRSA-[VI+fus] (1)	t362	FD	*fusC*	*seg, sei, egc gene cluster*	*agrI; cap8; sak/chp/scn; icaA/C/D*
CC59	ST59/952-MRSA-V(T) [PVL+], Taiwan Clone (1)	t437	K, E, CC, Cm	*ermB, aphA3, sat, cat*	*seb, sek, seq*	*agrI; cap8; chp/scn; icaA/C/D*
CC97	CC97-MRSA-V [fusC+] (4)	t267 (3), t359 (1)	Gm (4), K (4), E (1), CC (1), Te (1), FD (4), Cip (1)	*ermC, vgaA, aacA-aphD, fusC, tetK*	-	*agrI, cap5, Sak/scn, icaA/C/D*
CC80	CC80-MRSA-IV (1)	t8154	E, CC, Te	*ermC, tetK*	*etD; seb, sek, seq*	*agrIII; cap8; sak/chp/scn; icaA/C/D; edinB*
	CC80-MRSA-IV [PVL+], European caMRSA Clone (7)	t042 (2), t044 (3), t376 (1), t16186 (1),	Gm (3), K (7), E (1), CC (1), Te (3), FD (5), Cip (3)	*aacA-aphD, ermC, aphA3, sat, fusB, tetK*	*PVL; etD*	*agrIII; cap8; sak/scn; icaA/C/D; edinB*
CC88	CC88-MRSA-IV [PVL+] (1)	t12398 (1)	-	-	*PVL; sea (N315)*	*agrIII, cap8; sak/chp/scn; icaA/C/D*
	CC88-MRSA-IV, WA MRSA-2 (1)	t690	E, CC, Te	*ermC, tetK*	*sea (N315)*	*agrIII, cap8; sak/chp/scn; icaA/C/D*
CC121	CC121-MRSA-[V/VT+fus] (PVL+) (1)	t314	Gm, K, FD	*aacA-aphD, fusC, fosB,*	*PVL; seb, sei, egc gene cluster*	*agrIV; cap8; sak/scn; icaA/C/D*
CC152	CC152-MRSA-[V+fus] (PVL+) (2)	t355 (1), t11206 (1)	Gm (1), K (1), Te (1), FD (2)	*aacA-aphD, fusc, tetK*	*PVL*	*agrI; cap5; sak/scn; icaA/D; edinB*
CC361	CC361-MRSA-IV, WA MRSA-29 (1)	t3841	K, Tp, Cip	*msr(A), mphC, aphA3, sat, fosB*	*seg, sei, egc gene cluster*	*agrI; cap8; sak/scn; icaA/C/D*

CC	MRSA genotypes (N)	Spa types (N)	Antibiogram	Antibiotic resistance genes	Toxins	Miscellaneous
	CC361-MRSA-V, WA MRSA-70 (1)	t003	Gm, K	*aacA-aphD, fosB*	*sea (N315), seg, sei, egc gene cluster*	*agrI; cap8; sak/scn; icaA/C/D*
CC2250/2277	CC2250/2277-MRSA-IV, WA MRSA-114 (1)	ND	Tp	*fosB*	-	*sak/scn; icaA*
Singleton	ST2867-MRSA-V (1)	t148	-	*fosB*	-	*agrII; cap5; sak/scn; icaA/C/D; edinB*
	ST2867-MRSA-V/VT (1)	t148	-	*fosB*	-	*agrII; cap5; sak/scn; icaA/C/D; edinB*

Abbreviations: Cm, chloramphenicol; CC, clindamycin, Cip, ciprofloxacin; E, erythromycin; Fd, fusidic acid; Gm, gentamicin, Km, kanamycin; Te, tetracycline, Tp, trimethoprim; HLR-Mup, high-level mupirocin resistance.

*Two isolates (t127) each carried *ermC* and *sat* genes, while the one t127 isolate lacked *aphA3*.

**one isolate lacked *qacC*.

***one isolate carried *mupA* and *fusB* and lacked *ermC.*

****t306 isolate carried *aacA-aphD*, while t688 isolate carried *ermC*, *tetK*, *fexA.*

●one t311 isolate lacked *scn.*

●●t16185 isolate lacked *ermC*
